# Nano-Scaled Creep Response of TiAlV Low Density Medium Entropy Alloy at Elevated Temperatures

**DOI:** 10.3390/ma13010036

**Published:** 2019-12-20

**Authors:** Xiangkai Zhang, Hanting Ye, Jacob C. Huang, Taiyou Liu, Pinhung Lin, Yaocheng Wu, Mintsang Tsai, Yuchin Liao, Jason S. C. Jang

**Affiliations:** 1 Department of Materials Science & Engineering, Hong Kong Institute for Advanced Study, City University of Hong Kong, Kowloon, Hong Kong; kxzhang4-c@my.cityu.edu.hk; 2 Department of Materials and Optoelectronic Science, National Sun Yat-Sen University, Kaohsiung 804, Taiwan; pg21137@gmail.com (H.Y.); j199412383@gmail.com (T.L.); linnantou12345@gmail.com (P.L.); c830422@yahoo.com.tw (Y.W.); tommytsai0513@gmail.com (M.T.); 3 Department of Mechanical Engineering, Institute of Materials Science and Engineering, National Central University, Chung-Li 32001, Taiwan; llllurker@gmail.com (Y.L.); jscjang@ncu.edu.tw (J.S.C.J.)

**Keywords:** creep, low density medium entropy alloy, nanoindentation, hardness, activation energy

## Abstract

A low density, medium entropy alloy (LD-MEA) Ti_33_Al_33_V_34_ (4.44 g/cm^3^) was successfully developed. The microstructure was found to be composed of a disordered body-centered-cubic (BCC) matrix and minor ordered B2 precipitates based on transmission electron microscopy characterization. Equilibrium and non-equilibrium modeling, simulated using the Calphad approach, were applied to predict the phase constituent. Creep behavior of {110} grains at elevated temperatures was investigated by nanoindentation and the results were compared with Cantor alloy and Ti-6Al-4V alloy. Dislocation creep was found to be the dominant mechanism. The decreasing trend of hardness in {110} grains of BCC TiAlV is different from that in {111} grains of face-centered-cubic (FCC) Cantor alloy due to the different temperature-dependence of Peierls stress in these two lattice structures. The activation energy value of {110} grains was lower than that of {111} grains in FCC Cantor alloy because of the denser atomic stacking in FCC alloys. Compared with conventional Ti-6Al-4V alloy, TiAlV possesses considerably higher hardness and specific strength (63% higher), 83% lower creep displacement at room temperature, and 50% lower creep strain rate over the temperature range from 500 to 600 °C under the similar 1150 MPa stress, indicating a promising substitution for Ti-6Al-4V alloy as structural materials.

## 1. Introduction

Multi-principal element alloys (MPEAs), also termed as high-entropy alloys (HEAs) and medium-entropy alloys (MEAs), have been developed and researched extensively due to their promising mechanical properties, such as high strength [1], good wear resistance [2], and high corrosion resistance [3], since their first announcement in 2004 [4,5]. Classical MPEAs are mostly based on the FeCoNiCrMn, resulting in an average density ~8.1 g/cm^3^ [6,7]. More attention has been paid to the development of low-density multi-principal element alloys (LD-MPEAs) for energy-saving reasons [8,9,10,11,12,13,14,15,16,17,18,19]. Some of them are still single-phase solid solutions [14,15,16] and some are in dual-phase structures, and most of their densities are above 5.0 g/cm^3^, except for Al_20_Li_20_Mg_10_Sc_20_Ti_30_ [14]. However, the low melting points of Li and Mg could make applications at elevated temperatures a concern. It appears that the development of LD-MPEAs in either single-, dual-, or multiple-phased microstructures for applications at intermediate temperatures with densities less than 5.0 g/cm^3^ is still challenging. 

The nanoindentation technique has been extensively adopted to investigate the mechanical properties of solids with a small size. It has also been used for investigating the creep behavior of MPEAs at both ambient and elevated temperatures. Ma et al. [20] studied the creep behavior of face-centered-cubic (FCC) and body-centered-cubic (BCC) MPEA thin films at room temperature. They pointed out that creep strain of the FCC CoCrFeNiCu MPEA thin film increased by enhancing the holding load or loading rate, while a BCC CoCrFeNiCuAl_2.5_ MPEA thin film showed a better creep resistance. The creep behavior of grains with different orientations was studied in a single-phase FCC FeCoNiCrMn MPEA from room temperature to 600 °C in our previous study [21]. The dislocation-climb mechanism was dominant in both {111} and {100} planes, the hardest and the softest orientation, respectively. It is likely that the deformation kinetics in FCC structures could differ from those in BCC structures. It is, therefore, of interest to evaluate the creep behavior of the grains with the strongest nature, i.e., the {110} plane, in a BCC MPEA. To the best of our knowledge, the elevated temperature creep response of LD-MPEAs has not been reported. 

Ti and its alloys have wide applications in many fields, such as transportation and aerospace, because of their low density, corrosion resistance, and good medium temperature strength and creep resistance [22]. A classical Ti alloy is the Ti-6Al-4V (in wt.%) alloy which accounts for more than 50% applications of Ti alloys. However, Ti-6Al-4V alloy cannot be used at temperature higher than 400 °C due to its low strength at intermediate temperatures [22]. Hence, it is very attractive to develop alloys with comparable density with Ti-6Al-4V alloy but higher usage temperature.

Thus, in the present study, we performed nanoindentation experiments to explore the creep response of {110} grains in a BCC TiAlV LD-MEA from ambient temperature to 600 °C and compared that with a FCC Cantor alloy and conventional Ti-6Al-4V alloy. Additionally, the Calphad (representing CALculation of PHAse Diagrams) method, which has been widely used for the designing of MPEAs and prediction their phase composition and phase stability, was also carried out using Thermo-calc software employing the TTTi3 Thermodynamic Database.

## 2. Materials and Methods

A Ti_33_Al_33_V_34_ (in at%, thereafter TiAlV, or Ti_38_Al_21_V_41_ in wt.%) LD-MEA was prepared by arc-melting of the high-purity elements followed by drop casting. The purities of the starting elements are all greater than 99.9%. The casting process was conducted in high-purity argon atmosphere and the ingots were re-melted four times to ensure the uniform composition. The actual density of TiAlV is 4.44 g/cm^3^, quite close to its theoretical density 4.45 g/cm^3^ and much lower (45% lower) than the ~8.1 g/cm^3^ for the conventional MPEAs based on transition elements. Phases and lattice parameters were determined by the SIEMENS D5000 X-ray diffractometer (XRD, Karshrule, Germany) equipped with Cu-K*α* radiation. The operating voltage and current were set to be 40 keV and 30 mA, respectively. Scanning electron microscopy (SEM) was conducted by using the JEOL JSM-6330 TF field-emission scanning electron microscope (Tokyo, Japan), equipped with energy dispersive X-ray spectrometer (EDS). The grain orientations were revealed by using the gun Zeiss Supra 55 SEM (Oberkochen, Germany) with an (Electron backscatter diffraction) EBSD system. The sample size for SEM and EBSD characterization was 5 × 5 mm^2^, with 1 mm sample thickness. The flat sample surface was mechanically polished by 4000 emery paper, followed by electrochemical polishing. The area for EBSD orientation map construction is 350 μm × 250 μm. Crystal information is obtained from the EDAX Genesis analytical system computer software (OIM analysis 7.1, AMETEK, San Diego, CA, USA). A dual-beam focused-ion-beam (FIB) system (Seiko, SMI3050, Tokyo, Japan) was employed to make marks on the sample surface for position reference and prepare samples for transmission electron microscopy (TEM) analysis. The working voltage and ion beam current were set to be 30 keV and 1 pA. The TEM characterization was done on the field-emission TEM (Tecnai G20, FEI, Hillsboro, OR, USA) with an operating voltage of 200 kV.

The equilibrium phase diagram and non-equilibrium Scheil modeling was calculated using the Calphad method with the Thermo-calc Software (3.01, Thermo-Calc, Stockholm, Sweden) and the TTTi3 database. The nanoindentation creep tests were conducted in air and high-purity argon atmosphere at room temperature and elevated temperatures, respectively. The nanoindentation creep tests at 27, 400, 450, 500, 550, and 600 °C were carried out with a Hysitron TI Premier Nanoindenter (Bruker, Billerica, MA, USA) equipped with a sapphire Berkovich tip (Surface Technology, Hueckelhoven, Germany), operated with a peak load of 3000 μN, loading rate of 300 μN/s and holding time of 600 s under the load control mode. The details have been presented in [21]. Samples for nanoindentation creep testing were made by mechanical polishing, then by electrolytic polishing. Indentation creep measurements were all performed on the BCC {110} grains.

## 3. Results

### 3.1. Microstructures, Crystal Structure, and Calphad Calculation

**Figure 1:**
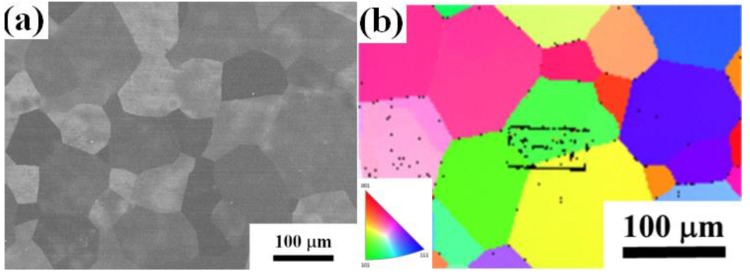
(**a**) SEM and (**b**) EBSD images of the TiAlV LD-MEA.

**Figure 2:**
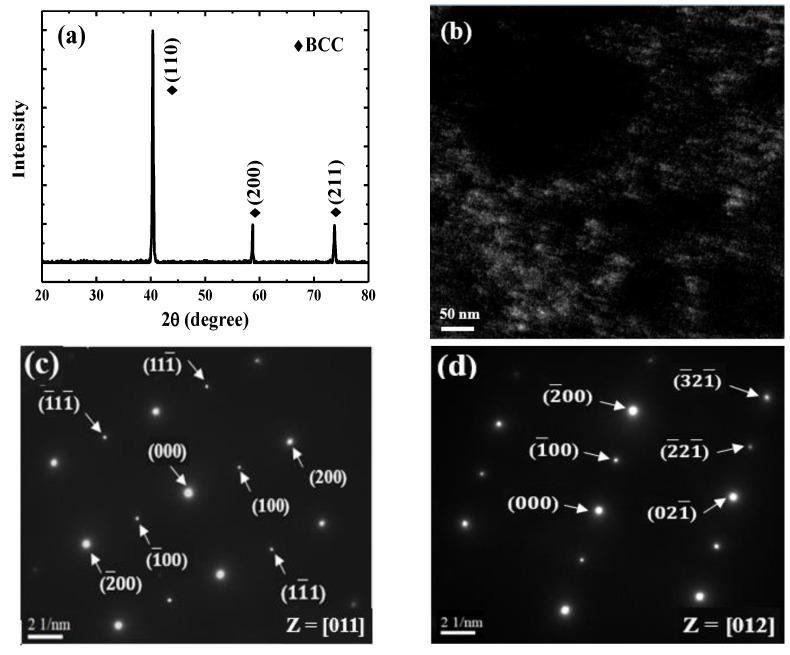
(**a**) XRD patterns of TiAlV LD-MEA; (**b**) TEM dark-field image; (**c**) and (**d**) corresponding SAED patterns.

**Figure 3:**
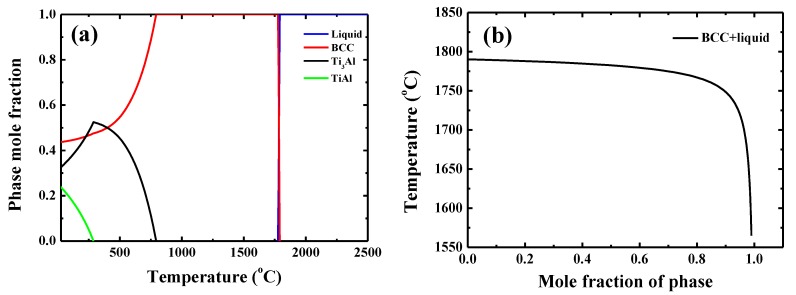
(**a**) Equilibrium phase diagram of TiAlV and (**b**) simulated non-equilibrium solidification curves for TiAlV LD-MEA (calculated by Thermo-calc with the TTTi3 database).

The typical microstructure of the TiAlV is shown in Figure 1a. All grains are equiaxed and the average grain size is identified to be about 80 μm. The contents of Ti, Al and V are 33 ± 2, 33 ± 1 and 34 ± 2 in at%, respectively. EBSD was carried out to determine the grain orientations and marks (black rectangles) were made by FIB for position reference to ensure the indenter tip is loaded on {110} grains interior, as shown in Figure 1b. Figure 2 shows the XRD patterns of the TiAlV LD-MEA. The as-cast TiAlV shows a single-phase BCC lattice structure. Note that the ordered B2 phase can often be found in Al-containing MPEAs [23,24,25]. The B2 phase may also exist in TiAlV but it cannot be detected by XRD due to its small volume fraction and/or small size, so the TEM characterization was performed. A nano-scaled phase (bright spots) was seen in the disordered BCC matrix, as seen in the TEM dark-field image in Figure 2b. The selected area electron diffraction (SAED) patterns confirm that these precipitates are the B2 phase. The simulated equilibrium phase diagram of TiAlV is presented in Figure 3a. Solidification starts at T_liq_ = 1790 °C and ends at T_sol_ = 1775 °C, with a solidification range of 15 °C. The BCC phase appears first after solidification begins. Then, with decreasing temperature, at the decomposition temperature T_dec_ = 790 °C, some of the primary BCC phase is predicted to partially transform to Ti_3_Al and then to TiAl phase at 280 °C. The temperature dependence of phase fraction during solidification of TiAlV is shown in Figure 3b. The non-equilibrium solidification modeling was performed using the Scheil model which is based on three assumptions: (i) no diffusion is in the solid; (ii) diffusion in the liquid is considered to be infinite; and (iii) equilibrium at the interface of the solid and liquid phases is kept [26]. Solidification of the studied alloy starts at 1790 °C and ends at 1565 °C, with the solidification range of 225 °C. A single BCC phase is predicted after solidification.

### 3.2. Creep Behavior

**Figure 4:**
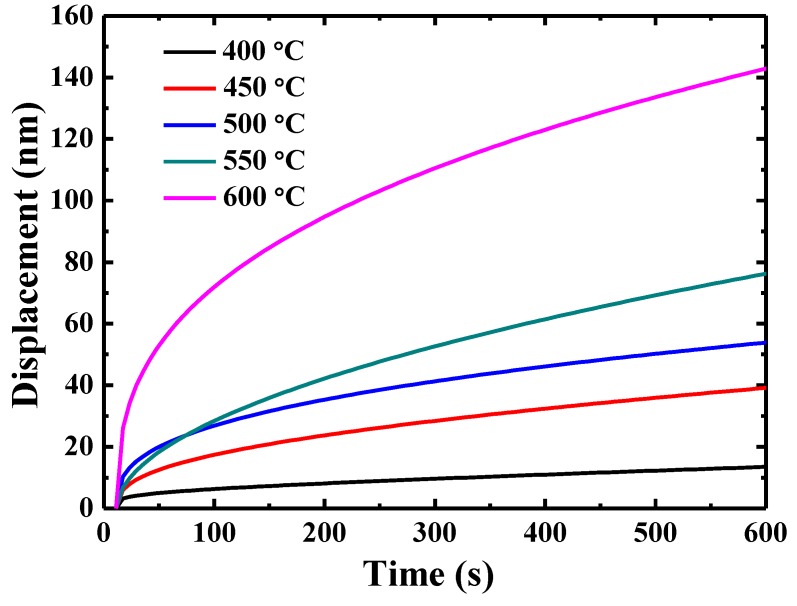
Creep displacement-holding time curves for {110} grains in TiAlV LD-MEA at different temperatures.

The representative creep displacement-holding time curves at different temperatures are shown in Figure 4. The indenter displacement increases dramatically at the beginning and then reaches a plateau when the testing temperature is 400 °C. In contrast, the creep curves for the testing temperature higher than 450 °C show an increasing trend, rather than a steady stage at the end of the creep curve. 

**Figure 5:**
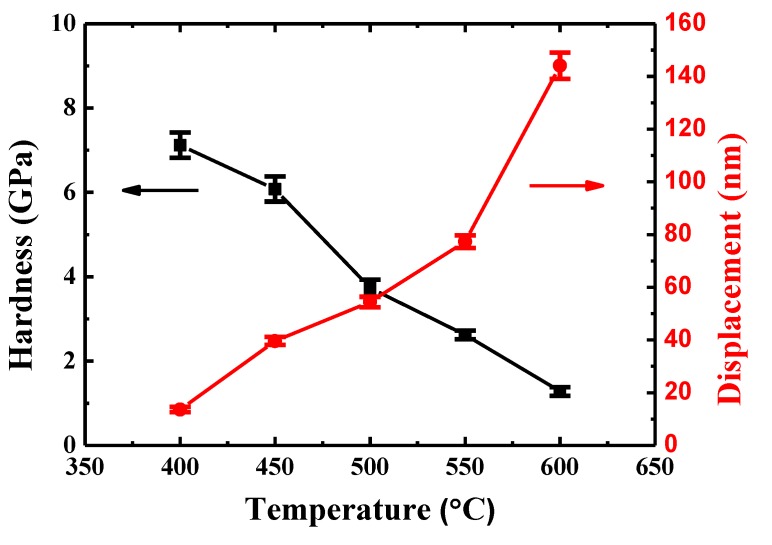
The hardness of {110} grains and indenter displacement in TiAlV as a function of temperature for a holding time of 600 s.

The creep displacements and hardness of {110} grains in TiAlV for a holding time of 600 s are given in Figure 5. It can be seen that the indenter displacement increases with temperature rising. More specifically, it was 13.6 nm at 400 °C and increased to 144.1 nm at 600 °C. The larger displacement at a higher temperature is expected because creep is a thermally-controlled process. The hardness of {110} grains in TiAlV almost maintains a constant value (~7.2 GPa) when the temperature increases from 27 to 400 °C, implying negligible creep deformation. Then, the hardness declines significantly, reaching a hardness of 1.3 GPa at 600 °C, which is in accord with the results of creep displacements.

**Figure 6:**
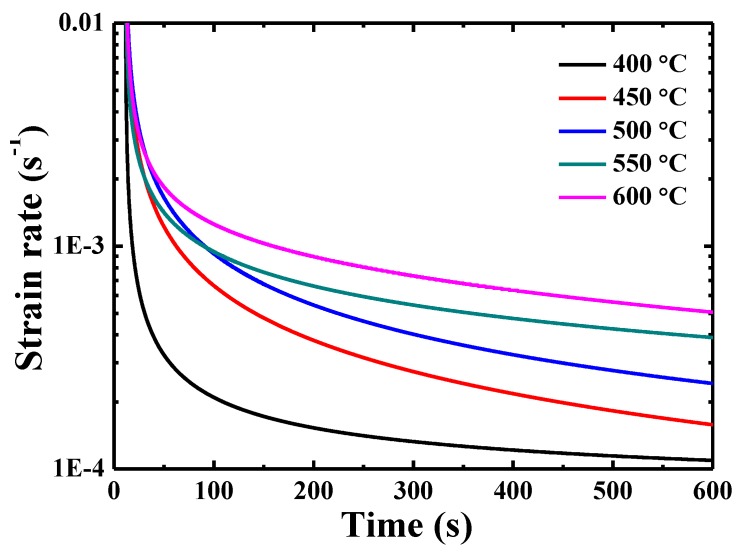
Curves of indentation creep rate-holding time for {110} grains in TiAlV LD-MEA.

Figure 6 presents the curves of indentation creep rate $\dot{\varepsilon }$ε—holding time. The indentation creep rate can be obtained by using the following equation [27]:

(1) $$\dot{\varepsilon }=\frac{d\varepsilon }{dt}=\;\frac{1}{h}\left[\frac{dh}{dt}\right],$$

where *h* and *t* are the total indenter displacement and holding time, respectively. Two distinct stages can be seen: transient state and quasi-steady state, where the creep rate first decreases rapidly then tends to reach an obvious quasi-steady state with a creep rate varying from 1.08 × 10^−4^ s^−1^ at 400 °C to 4.98 × 10^−4^ s^−1^ at 600 °C. The strain rate at 600 s does not seem to be a quasi-steady state, which is a compromise among the creep behavior, thermal drift and oxidation. 

The strain rate sensitivity (SRS) *m* can be calculated by the equation:$\;\sigma =A{\dot{\varepsilon }}_{f}^{m}$ where *σ*, *A* and $\dot{\;\varepsilon_{f}}\;$are flow stress, pre-coefficient and creep rate at the final testing time. The flow stress *σ* can be obtained by using the empirical formula: *H = Cσ*, where *H* is the hardness and *C* the constraint factor which is typically ~3 for metals [28]. 

The results are summarized in Table 1. The average value of stress exponent *n* (*m* = 1/*n*) is ~4.1 over 400–600 °C in TiAlV. It is well known that the *n* is a useful parameter for estimating the creep mechanisms such that *n* = 1 for diffusional creep, *n* = 2 for the grain boundary sliding (GBS), and *n* between 3 and 8 for dislocation glide plus climb [29,30]. The *n* values for {110} grains in TiAlV fall in the range of 3–8 over the temperature range between 400 and 600 °C, suggesting that the dominant creep mechanism is dislocation glide plus climb. It was reported that GBS accommodated by dislocation glide and climb can also have *n* values ranging from 2 to 5 [31,32]. The current alloy has a large grain size, about 80 μm, and the creep tests were done in the center of the {110} grains, thus GBS is unlikely to occur.

**Figure 7:**
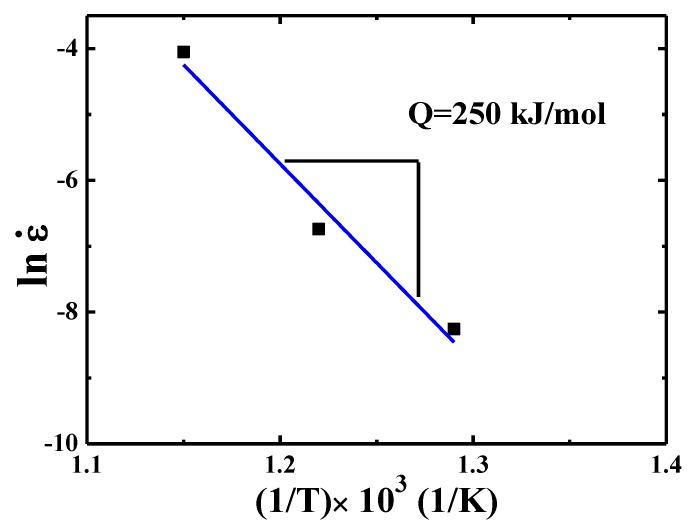
Strain rate versus 1000/T for {110} grains in TiAlV LD-MEA.

By plotting the curve of creep strain rate as a function of flow stress σ, the activation energy *Q* can be extracted. Similar to the analysis presented in Ref. [21], under a fixed normalized stress *σ/E* (where *E* is the temperature dependent Young’s modulus) of 1.28 × 10^−2^, the effective strain rate ${\dot{\varepsilon }}_{eff}$ corresponding to this fixed stress *σ*/*E* at different temperatures can be obtained. The results are summarized in Table 1. Then the *Q* can be evaluated according to the equation [34]:

(2) $${\dot{\varepsilon }}_{eff}=B\left(\frac{\sigma }{E}\right)nexp\left(\frac{-Q}{RT}\right),$$

where *B* and *R* are a pre-coefficient related to grain size and gas constant, respectively, as presented in Figure 7. The *Q* value is 250 kJ/mol for {110} grains over 500–600 °C.

The activation volume *V* can shed light on the thermally activated mechanism during the creep and can be calculated by the following equation [35]:

(3) $$V=\frac{\sqrt{3}k_{B}T}{m\sigma },$$

where *k_B_* is the Boltzmann constant. The results are listed in Table 1. The *V* value increases with increasing temperature, indicating that the volume involved in vacancy diffusion increases at high temperature. Specifically, the values of *V* for {110} grains in TiAlV increases from ~2 *b*^3^ (*b* is the Burger’s vector) and ~2 Ω (Ω is the atomic volume) at 27 °C to ~10 *b*^3^ and ~13 Ω at 600 °C, respectively. This can be predicted since in MPEAs, a vacancy is surrounded by various alloying elements and the exchange between vacancy and atoms is more complex, which involves cooperative movement of several atoms to maintain proper composition portioning [21] and, thus, an increment of activation volume. 

## 4. Discussion

### 4.1. Comparison of the Thermodynamic Calculations with Experimental Data

For the equilibrium phase diagram, although three phases, BCC, Ti_3_Al, and TiAl, were predicted, and the experiment observation shows that the BCC and B2 phase formed in the as-cast TiAlV. Additionally, the equilibrium phase diagram can be used to predict the phase formation in MPEA. Gao et al. [36] pointed out that the ratio of the temperature range in which the primary phase is stable over the solid temperature, i.e., (T_sol_−T_dec_)/T_sol_, is larger than 0.3, a single-phase solid solution would form in the as-cast MPEA. In the case of as-cast TiAlV, the ratio is 0.55 by using the T_sol_ = 1715 °C and T_dec_ = 790 °C determined above and a single-phase BCC solid solution is expected. However, the B2 phase appeared, indicating that the criterion is invalid for the TiAlV.

The non-equilibrium solidification modeling predicted the formation of a single-phase solid solution in the as-cast TiAlV without the presence of B2 phase which was observed experimentally. It can be concluded that the Thermo-calc software using the TTTi3 database can predict correct trends, but does not provide an accurate phase constitution. The reasons for the formation of BCC lattice structure in TiAlV may be explained in the following way: The lattice structure of V is BCC and Ti has BCC lattice structure at temperatures higher than 882 °C [37]. Although Al possess FCC lattice structure, it was found not only in other MPEA systems such as Al_x_CoCrFeNi but also steels that Al serves as a strong BCC stabilizer [38,39]. The BCC phase forming at high temperatures was very stable and was retained after the drop casting process, although a minor B2 phase formed. In addition, the actual density measured by hydrometers is very close to its theoretical density ρ estimated by rules of mixture, i.e., $\rho =\frac{\sum^{\text{​}}c_{i}M_{i}}{\sum^{\text{​}}c_{i}V_{i}}$, where $c_{i},M_{i}\;and\;V_{i}$ are the atomic fraction, molar mass and molar volume, respectively. This indicates that no intermetallic phases formed, otherwise the error can be high [40].

### 4.2. Comparison of the Creep Behavior of TiAlV LD-MPEA with Cantor Alloy

Due to the deformation kinetics difference between the FCC structures and BCC structures, it is worth comparing the creep behavior in the strongest {111} plane of FCC and the strongest {110} plane of BCC.

**Figure 8:**
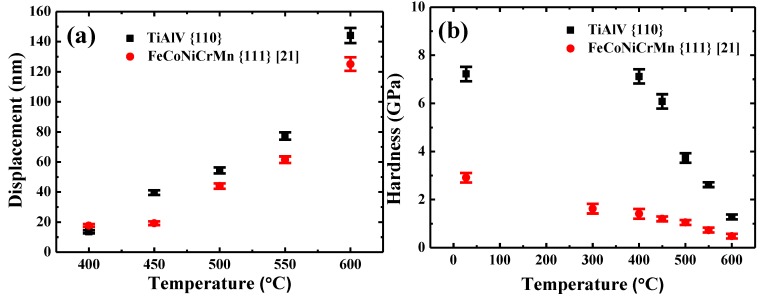
(**a**) The indenter displacement and (**b**) hardness of {110} grains in TiAlV LD-MEA and {111} grains in FeCoNiCrMn HEA [21] as a function of temperature.

Figure 8a compares the indenter displacements in {110} grains of TiAlV LD-MEA and {111} grains of FeCoNiCrMn HEA [21]. Note that the indenter displacement at 400 °C for {110} grains in TiAlV is slightly less than that for {111} grains in FeCoNiCrMn, suggesting that {110} grains in TiAlV has a higher creep resistance at 400 °C. However, at higher temperatures above 450 °C, the creep displacement becomes higher than that of FeCoNiCrMn, indicating the lower creep resistance of TiAlV at higher temperatures. The hardness of {110} grains of TiAlV and {111} grains of FeCoNiCrMn versus temperature is shown in Figure 8b. The hardness of {110} grains in TiAlV is consistently higher than that of {111} grains in FeCoNiCrMn. Although the hardness data all decrease, they exhibit a different decreasing trend. Specifically, the hardness of {110} grains in TiAlV almost maintains a constant value (~7.2 GPa) when the temperature increases from 27 to 400 °C, implying negligible creep deformation. Then, the hardness declines significantly, reaching a hardness of 1.3 ± 0.1 GPa at 600 °C. In contrast, the hardness of {111} grains in FeCoNiCrMn shows a continuously decreasing trend from 2.9 ± 0.2 GPa at 27 °C to 0.5 ± 0.1 GPa at 600 °C. The major softening at elevated temperatures is caused by the diffusion phenomenon [41].

The different hardness decreasing trend in TiAlV and FeCoNiCrMn may be explained as follows: It is known that the flow stress is comprised of two parts: the athermic part and the thermal part, i.e., Peierls stress, which is temperature dependent. The contribution of the Peierls stress to the flow stress vanishes when the temperature reaches a critical temperature T_c_ [42]. In the current BCC TiAlV, the T_c_ was estimated to be 400 °C beyond which a significant decrease was observed from 7.12 at to 6.08 GPa at 450 °C. For the FCC FeCoNiCrMn, however, although the Peierls stress cannot be ignored at temperature below room temperature, it is negligible at elevated temperatures because the height of the Peierls barrier decreases dramatically [43]. This trend in FCC MPEAs also observed by Otto et al. [43] and Wu et al. [44].

The activation volume for Cantor alloy was reported to be 2 *b*^3^ at room temperature and 4–8 *b*^3^ at 400–600 °C [21,45], which is the same order as the present alloy. The *Q* value is 250 kJ/mol for {110} grains over 500–600 °C, which is a little lower than that obtained in {111} grains of FeCoNiCrMn (259 kJ/mol) [21]. Coupled with the lower creep displacement shown in Figure 8a, and the higher activation energy *Q* for FeCoNiCrMn, it is ensured that the creep resistance of {111} grains in FeCoNiCrMn over 450–600 °C is better than that of {110} grains in TiAlV. This may be attributed to the fact that the self-diffusivity in alloys with FCC structures is usually lower than that in alloys with BCC structures due to a higher atomic stacking density in FCC structures [46].

### 4.3. Comparison of the Creep Behavior of TiAlV LD-MPEA with Ti-6Al-4V Alloy

It was reported that the creep displacement of commercial Ti-6Al-4V alloy (in wt.%) is about 17.5 nm with a holding time of 200 s and holding load of 3000 μN at room temperature [33]. In comparison, the displacement of current TiAlV with the same holding time and load is only 8.1 nm (54% lower than that in Ti-6Al-4V alloy) even at 400 °C, which is the upper limitation temperature for industry applications of Ti-6Al-4V alloy [22]. A separate run for TiAlV at room temperature reveals a creep displacement of only 3 nm (83% lower than that in Ti-6Al-4V alloy), demonstrating a superior creep resistance of TiAlV than that of Ti-6Al-4V alloy at room and intermediate temperatures. In addition, the hardness normalized by density, or the specific hardness, of TiAlV is 1.6 GPa/g/cm^3^, which is considerably higher (by 63%) than that of Ti-6Al-4V alloy (0.98 GPa/g/cm^3^) [33], as compared in Table 1. 

**Figure 9:**
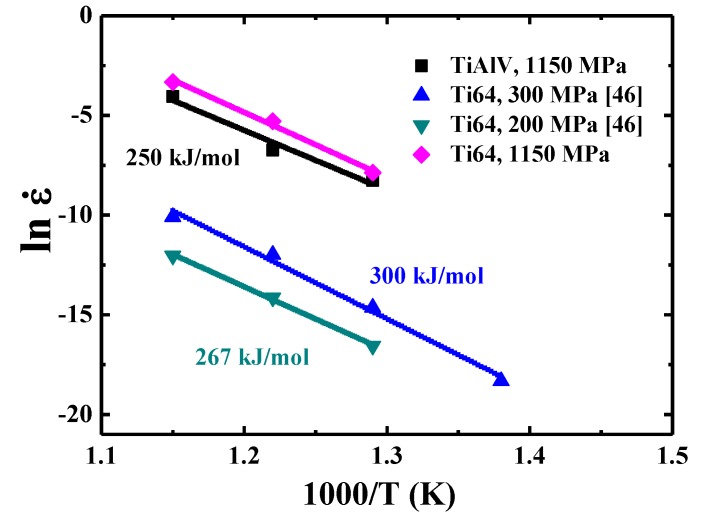
Strain rate versus 1000/T for TiAlV LD-MEA and Ti-6Al-4V alloys [33].

A large range of creep activation energy values for Ti-6Al-4V alloys with different microstructures and testing conditions were reported, from 185 to 350 kJ/mol [47,48,49,50]. The *Q* value (250 kJ/mol) of TiAlV is comparable to those of Ti-6Al-4V alloys. An attempt has been made to compare the strain rate level of TiAlV and Ti-6Al-4V alloy under the same load over the temperature range of 500–600 °C. However, the creep testing for the bulk sample of Ti-6Al-4V alloy was usually conducted under much lower load, for example, 200–300 MPa [50], as shown for two examples in Figure 9. In order to compare with the loading (around 1150 MPa) for TiAlV under nanoindentation, the extrapolation method is applied by using the equation $\sigma =A{\dot{\varepsilon }}_{f}^{m}\;$for Ti-6Al-4V alloys with *n* ~5 or *m* ~0.2. It is found that the resulting creep strain rate (as if Ti-6Al-4V alloy were tested under nano-scaled nanoindentation creep) would be much higher under a load of 1150 MPa. The strain rates for Ti-6Al-4V alloys under 1150 MPa are also plotted in Figure 9 for comparison, all higher than those of TiAlV LD-MEA under the same temperature and applied stress levels by about 50%.

## 5. Conclusions

In the current study, a TiAlV LD-MEA (4.44 g/cm^3^) was successfully developed. The TEM results revealed that a small amount of ordered B2 particles in the disordered BCC matrix. The Calphad method was employed to calculate equilibrium and non-equilibrium solidification modeling using Thermo-calc software and the TTTi3 database. The non-equilibrium solidification modeling can predict correct trends, but does not guarantee a precise phase constitution.

The nanoindentation tests were conducted to investigate the creep behavior of {110} grains up to 600 °C. The experiment results were compared with FeCoNiCrMn MPEA and Ti-6Al-4V alloy. The different decreasing trend in hardness in {110} grains of TiAlV LD-MEA and {111} grains of FeCoNiCrMn HEA with increasing temperature results from the negligible contribution of Peierls stress in FCC lattice structure. The better creep resistance of {111} grains in FeCoNiCrMn HEA over 450–600 °C may be ascribed to a higher atomic stacking density in FCC lattice structure. The activation energy value of {110} grains was estimated to be 250 kJ/mol, which is lower than that of {111} grains in FCC FeCoNiCrMn HEA because of denser atomic stacking in FCC lattice structure. In comparison with commercial Ti-6Al-4V alloy, TiAlV LD-MEA possesses much higher room temperature hardness and specific strength (63% higher), much lower creep displacement at room temperature (83% lower), and 50% lower creep strain rate over the temperature range between 500 and 600 °C under the similar 1150 MPa stress, suggesting greater creep resistance than the Ti-6Al-4V alloy. Therefore, TiAlV LD-MEA appears to a promising substitution for Ti-6Al-4V alloy as structural materials used over this elevated temperature range. 

## Figures and Tables

**Table 1:** Summary of the experimentally measured and extracted nanoindentation data on the {110} grains in TiAlV LD-MEA, as well as FeCoNiCrMn HEA and Ti-6Al-4V alloy. Strength data are converted from hardness values divided by 3.

*T*, °C	*n*	*E*, GPa	σ*_f_*, MPa	σ*_f_*/E	${\dot{\varepsilon }}_{f}{,}_{\;}s^{-1}$	${\dot{\varepsilon }}_{eff},\;s^{-1}$	*V,* Ω
400	4.1	126 ± 4	2374 ± 100	1.88 × 10^−2^	1.09 × 10^−4^	2.25 × 10^−5^	2
450	4.1	114 ± 4	2026 ± 100	1.78 × 10^−2^	1.55 × 10^−4^	4.02 × 10^−5^	2
500	4.1	99 ± 4	1243 ± 67	1.26 × 10^−2^	2.38 × 10^−4^	2.59 × 10^−4^	4
550	4.1	90 ± 3	872 ± 33	9.69 × 10^−3^	3.84 × 10^−4^	1.18 × 10^−3^	6
600	4.1	80 ± 3	428 ± 33	5.35 × 10^−3^	4.98 × 10^−4^	1.74 × 10^−2^	13
***T*, °C**	**Alloy**		***H*, GPa**	**σ*_f_*, MPa**		***T_m_*, ^o^C**	**Ref.**
RT	TiAlV		7.2 ± 0.1	2400 ± 100		1418	-
RT	FeCoNiCrMn		2.9 ± 0.2	967 ± 100		1528	[21]
RT	Ti-6Al-4V		4.4	1467		1668	[33]
